# The practical, ethical and legal reasons why patients should not be transferred between NHS trusts for phage therapy

**DOI:** 10.1177/25160435221120300

**Published:** 2022-08-09

**Authors:** Joshua D Jones, Pamela R Ferguson, Mehrunisha Suleman

**Affiliations:** 1 Infection Medicine, 523476 Edinburgh Medical School: Biomedical Sciences, University of Edinburgh, Edinburgh, UK; 2 School of Law, Scrymgeour Building, 3042 University of Dundee, Dundee, UK; 3 Nuffield Department of Population Health, Ethox Centre, 6396 University of Oxford, Oxford, UK

**Keywords:** Safe practice

## Abstract

Bacteriophages (phages) are naturally occurring viruses of bacteria that have a long history of use as antimicrobials, known as phage therapy. The antibiotic resistance crisis has driven renewed interest in phage therapy, which has been used on an unlicensed compassionate basis in various Western contexts. The option to use unlicensed medicines exists to allow clinicians to respond to genuine clinical needs arising in their own patients. However, in the UK some clinicians may, in the absence of suitable patients of their own, seek to transfer patients from other NHS trusts into their own Trust. This article sets out why patient transfer is not necessary and the practical, ethical and legal reasons why patients should not be transferred between NHS Trusts for phage therapy. Phage preparations should always be transported to the patient and the patient treated in the Trust in which they would have received care in the absence of phage. We enclose suggested best practice guidelines for adoption across the UK that will protect patient safety and safeguard clinicians and Trusts from potential litigation.

## Background

Bacteriophages (phages) are naturally occurring viruses of bacteria that infect bacteria in a species-, and sometimes even strain-, specific manner. There are an estimated 10^31^ phages on the planet and more phages on/in you than cells in your body.^1,2^ Discovered in the early 20th century, phages can be used to treat bacterial infection, known as phage therapy, in which phages infect and lyse bacteria at the site of infection.^3^ Whereas traditional antibiotics are chemotherapeutic agents, phage are anti-bacterial biological agents. Phage therapy has a long and curious history, with the first report of its use dating back to 1919.^4^ There followed widespread use in the ‘West’, largely curtailed by the mass-production of antibiotics in the 1940s. Meanwhile, phage therapy persisted in the ‘East’, notably Russia, Georgia and Poland.^5^ Phage therapy can be delivered in either pre-formulated phage cocktails, targeting a range of bacterial species, or a patient’s bacterial isolate can be tested against a collection of phages to generate a personalised phage cocktail.

Modern medicine relies on antibiotics to treat bacterial infection. However, bacteria can become resistant to antibiotics. A recent report estimated that globally 4.95 million deaths were associated with antibiotic resistance in 2019 and it has been estimated that by 2050 antibiotic resistance could kill 10 million people annually.^6,7^ The spectre of antibiotic resistance has therefore driven interest in alternative antibacterial strategies. Phage therapy represents a promising alternative antibacterial strategy which enables the treatment of patients with antibiotic resistant infections and there is evidence that some combinations of phages and antibiotics can be synergistic.^8,9^ Phage therapy is therefore currently experiencing a renaissance and there have been an increasing number of clinical applications throughout the ‘West’ among patients whose clinical needs are not met by antibiotics, for example due to antibiotic resistance.^10,11^ Phage therapy is not yet licensed in Western nations and can currently only be used on a unlicensed compassionate basis, generally as an adjunct to antibiotics. Practically, a suspension of phage particles is administered to the site of infection; this can be by any route, for example topical, nebulised or intravenous.^8^ In the UK, the first author has recently overseen the provision of adjunctive topical anti-staphylococcal phage therapy to 10 diabetic foot infection patients at high risk of amputation despite receiving appropriate antibiotics at hospitals in Edinburgh and Glasgow (unpublished, 2022). Elsewhere, two paediatric cystic fibrosis patients have been treated with intravenous anti-mycobacterial phage therapy at Great Ormond Street Hospital.^12,13^ These applications are supported by promising and reassuring literature around the safety and efficacy of phage therapy.^8,11^

In the UK, the use of unlicensed medicines exists for when, in the opinion of the patient’s clinician(s), licensed treatments are not meeting the patient’s clinical needs and it is in the patient’s best interest to explore unlicensed options. Patients for whom phage therapy may be appropriate include those with: wholly antibiotic resistant infections; antibiotic susceptible but clinically recalcitrant chronic infections; reasonably foreseen acute risk to life or limb despite appropriate antibiotic treatment; other patient-specific factors that preclude the use of appropriate antibiotics (e.g. renal failure or allergy) or mean that further medical intervention is preferred to surgery (e.g. high-risk surgical candidate). This represents a sizeable number of patients across the NHS. For example, there were 29,695 major or minor amputations for UK patients with diabetes between the three years of 2017/8 and 2019/20.^14^ Although infection is not the only cause of diabetic foot amputations it is a significant cause and many of these patients may have had infections treatable by phage therapy. Orthopaedic infections, particularly chronic prosthetic joint infections, may also benefit from phage therapy. For example, the UK National Joint Registry 18th Annual Report shows that over the last 5 years there have been 6804 hip and 7919 knee primary and secondary revisions due to infection.^15^ As phage therapy becomes more widespread in the UK, consideration needs to be given to how it can be best delivered in a way that centres on patients’ best interests. The NHS in the UK is divided into ‘Trusts’, these are the organisational units of the NHS and generally provide healthcare for defined geographical portions of the UK. The long-term vision in the UK is a distributed model in which patients receive phage in their own Trust, with most receiving off-the-shelf cocktails and personalised therapy for a minority provided remotely by a national specialist centre. This will serve the needs of patients equitably and stimulate the development of uniform clinical practices. Crucially, the provision for the use of unlicensed medicines exists to allow clinicians to respond to genuine clinical needs arising in their own patients. However, some clinicians may, as illustrated by the real-world example in Box 1, in the absence of suitable patients of their own, seek to transfer patients from other NHS trusts into their own Trust. This article sets out the practical, ethical and legal reasons why patients should not be transferred between NHS Trusts for phage therapy, summarised in Table 1.

Box 1. A real-world example of clinicians seeking unjustified patient transfer for phage therapy.

Clinicians from a particular specialty in a Trust recognised the potential value of phage from literature and conference attendance. However, these clinicians had no clinical needs arising from their own patients for which the use of unlicensed phage might be appropriate. Nonetheless, wanting to gain experience with phage to develop themselves as a ‘centre of expertise’ in phage therapy for their specialty, the clinicians attempted to identify potential patients from other Trusts. Despite not having a source of phage, they claimed ‘expertise’ in phage therapy and responded to requests for help with complex patients from other Trusts. Having identified a potential patient in another Trust, the clinicians neglected to inform either the patient or the patient’s own clinician that the patient could access phage in their own local Trust, without any involvement of another Trust. Despite the patient’s own Trust having a comprehensive unlicensed medicines policy, the clinicians argued that their Trust, with a comparable policy, had better governance in place. Despite the patient’s local clinician becoming aware that patient transfer was unnecessary, the clinicians proceeded to arrange a consultation with the patient to discuss their transfer to and treatment in their Trust.

*This raised serious concerns, set out in this article, and unnecessary patient transfer was averted in this case. However, the clinicians continued to pursue further unjustified patient transfers.*

**Table 1.** Reasons why patients should not be transferred between NHS trusts for phage therapy and best practice guidance for preventing unjustified transfers.

Reasons why patients should not be transferred between NHS Trusts for phage therapy	
Clinical expertise	There is no practical specialty-specific clinical expertise required for the administration of phage therapy, the necessary clinical skills exist in all Trusts.
Clinical microbiology expertise	Clinical phage therapy requires specialist clinical microbiology input in the form of *in vitro* assays and the provision of general advice to clinicians. This can be undertaken remotely.
Availability of phage	Phage is not readily available, but all Trusts are equally able to source phages if needed.
Governance	All Trusts have appropriate policies and governance mechanisms to enable the use of unlicensed phage.
Patient safety	There are inherent risks associated with patient transport. It is inconceivable that it would ever be in the best interest of patients for them to be moved to the source of phage rather than couriering phage to the patient’s own Trust.
Patient trust	Unnecessary patient transfers could suggest a Trust was exploiting vulnerable patients for their own gain. This would irreparably damage broader patient trust in valuable unlicensed medicines, including phage.
Legal action	Unnecessary patient transfer carries legal risks. Patient harm arising could warrant medical negligence claims. A criminal case for an unauthorised clinical trial could be made against Trusts ‘recruiting’ patients. A Trust would breach its legal obligation under the NHS Constitution if it were offering patients from other Trusts the ‘choice’ to receive phage in their Trust.
Best practice guidance for preventing unjustified patient transfers	
Patients should be treated in the Trust they would have been seen in had phage not been available	
Phage must always be transported to the patient, not the other way around	
As part of governance, it should be confirmed that the patient’s home address lies within the remit of the treating Trust	

## Why patients should be treated in their own NHS trust

There are several practical reasons why potential phage patients should be treated in their own NHS Trust (Table 1). First, all NHS Trusts can and do use unlicensed medicines. In this respect phage is no different and the existing governance structures which cover these activities in all Trusts cover phage as well. Second, the administration of phage is practically straightforward and does not require any specialist clinical skills not already available in all Trusts. For example, in the UK phage has so far been administered to patients by intravenous, intraperitoneal and topical routes.^8^ Any clinicians claiming clinical expertise should therefore be met with scepticism. Third, although access to phage across Europe is currently on an ad hoc basis and driven by networking between clinicians and clinical phage laboratories, clinicians from all Trusts are equally able to pursue these avenues should they find themselves confronted by a patient whose clinical needs cannot be met by antibiotics and in whose best interests it might be to explore unlicensed treatment options.

Although we have just established that all Trusts are equally able to pursue phage, if theoretically only one Trust had access to phage therapy it would be inconceivable that it would ever be in the best interest of patients for them to be moved to the source of phage. This is because, as mentioned above, the administration of phage requires no specialist clinical expertise, so can be used in any Trust, and the phage can be posted to any Trust. This is even more implausible given that patients with chronic infections may have ongoing issues (e.g. mobility or frailty) that make travel unsuitable or mean that travel exposes them to additional and unnecessary risk and it is similarly unlikely that it would ever be appropriate to transport acutely unwell patients. The transport of patients in this way would also likely and unnecessarily take patients away from their support networks, such as friends and family. Because of the default to specialisation, it is regrettable that the scenario in Box 1 may be repeated in future. We must conceptualise phage in the same way we would a new type of antibiotic, which would be available to all Trusts and for which patient transfer would never be considered. As illustrated in Box 1, Trusts therefore seeking transfers of potential patients on the grounds of access to phage, improved governance or clinical expertise would be making spurious claims. To transfer a patient on such a basis is also unethical and leaves the Trust legally exposed, as we will explore later (Table 1).

All potential patients must be made aware that access to, and the clinical expertise required for, phage is equally available in their own Trust and there is therefore no need for them to be transferred to a different NHS Trust to receive phage. Importantly, the ‘courts routinely have regard not just to professional norms and standards but also to patients’ rights and the core needs and entitlements those rights protect, in developing medical law’.^16^ One of the leading legal decisions in the UK is Sidaway v Governors of Bethlam Royal Hospital.^17^ Decided in 1985 by the House of Lords, this case made clear that healthcare professionals owed a duty to their patients to disclose risks which were ‘so obviously necessary to an informed choice on the part of the patient’ that no ‘prudent’ doctor would fail to disclose them. Clearly since no doctor should expose their patient to unnecessary risk, any decision to transfer a patient from one Trust to another would need to be justified and the reasoning clearly explained to the patient during the informed consent process. Moreover, ambitious clinicians must not exploit professional relationships to persuade colleagues in other Trusts to permit unnecessary patient transfer by neglecting to make it clear to a patient that there is no need to transfer them. To do so would prevent that patient giving fully informed consent and place the patient at unnecessary risk of harm during patient transfer. From a legal perspective, providing a patient with inadequate informed consent would create a case for negligence. It is inconceivable that any fully informed patient would choose to travel unnecessarily outside of their local area to receive a treatment that could have been administered in their local Trust.

It is also not possible for patients to simply choose to receive phage therapy in any Trust. The actions of a Trust attempting to justify inter-Trust patient transfer on the grounds of patient choice are contrary to the NHS constitution and NHS Choice Framework, which only permit patient choice of hospital at the point of first outpatient referral.^18^^,19^ This makes sense from the perspective of the NHS being composed of distinct NHS Trusts, otherwise the best hospitals would be overrun by demand from across the country. All NHS Trusts have a legal obligation, under the Health Act 2009, to have regard to the NHS Constitution.^20^

Let’s suppose for a moment that a less-than-fully-informed patient agrees, travels to another Trust and undergoes a surgical procedure during which phage is administered. This creates scenarios that are both likely and troubling. First, let’s suppose that the patient, already being clinically vulnerable and likely to be of an older demographic, dies during the operation. In England intraoperative deaths are ‘deaths of immediate concern’ and investigated as ‘serious untoward incidents’, potentially involving referral to a coroner.^21^ This leaves the receiving Trust in the position of having to explain why a clinically vulnerable patient from a different Trust was receiving an unlicensed treatment in their Trust that could have been accessed and administered within that patient’s own Trust. The subsequent inevitable investigation will raise serious legal and ethical questions, not least of these will be whether the receiving Trust has recruited, or more arguably exploited, a vulnerable patient to further its own ambitions and whether this represents a lack of care on the part of the receiving Trust. The Trust who receives the patient assumes a duty of care to that patient and may be sued under the tort (in England and Wales) or delict (in Scotland) of negligence if the standard of care which is given falls below that of the ‘reasonable doctor’ – as it surely would if the patient ought not to be treated in the receiving trust or is harmed in transit. A case for negligence in the standard of care could also be made against the ‘referring’ Trust. Moreover, given both the context of pre-existing ambition and especially the potential for concurrent data collection, the actions of the receiving Trust in this scenario could potentially appear to take the form of an unregulated clinical trial, which, if sustained, would be a criminal offence under The Medicines for Human Use (Clinical Trials) Regulations 2004.^22^

Aside from the legal implications, the public and media perception of any Trust engaging in unjustified transfers is likely to be that the Trust was exploiting vulnerable patients for their own gain. Such a public perception would cause enormous damage to the reputation of phage therapy in the public consciousness and undermine both confidence in valuable unlicensed medicines in general and also in phage therapy. Any future patients whose clinical needs are not met by antibiotics and are presented with the option of phage therapy could rightly be sceptical of clinicians’ motivations.

Ultimately, when phage therapy is deemed appropriate, the use of phage must centre on what is best for the patient. The phage preparation must always be transported to the patient in their local context and patients should not be moved between Trusts to receive phage therapy. The only exception to the ‘phage to patient’ rule is the scenario in which the patient would have been transferred between Trusts for specialist care not available in their own Trust, regardless of the availability of phage. It is therefore unlikely that there would ever be grounds to transfer patients from major specialties, such as orthopaedics, between Trusts, as almost all Trusts offer a full range of clinical expertise in these areas. While there is an onus on senior clinicians to place patient safety ahead of professional ambition, as a necessary safeguard we recommend that those involved in the oversight of unlicensed phage, for example pharmacists, in any Trust independently confirm that the patient’s home address lies within the remit of their Trust. If it does not, and the patient would not otherwise be treated in that Trust in the absence of phage, then the patient should be treated in their own Trust. It is not inconceivable that non-medical staff, such as pharmacists, may come under pressure from medical colleagues in this regard. This peer-reviewed article and best practice guidelines enclosed (Table 1) are therefore provided to support those involved in the governance of unlicensed phage across the UK.

## Conclusion

In summary, inter-Trust patient transfers for unlicensed phage are not justified because phage can be accessed, governed and administered by any Trust. This article has set out guidance to safeguard patients and clinicians involved in the use of phage across the UK. Phage must always go to the patient and patients must only be treated with phage in an NHS Trust in which they would already be receiving care. Failure to heed the arguments laid out here raises ethical and legal issues and exposes Trusts and individual clinicians to the risk of civil suit and criminal prosecution. It also is irresponsible in placing ambition above patient safety and may imperil the continued provision of, and confidence in, unlicensed phage therapy for patients whose clinical needs cannot be met by antibiotics in the UK.
